# High NAFLD fibrosis score in non-alcoholic fatty liver disease as a predictor of carotid plaque development: a retrospective cohort study based on regular health check-up data in China

**DOI:** 10.1080/07853890.2021.1974081

**Published:** 2021-09-09

**Authors:** Xinyan Yu, Chen Chen, Yi Guo, Yuling Tong, Yi Zhao, Lingyan Wu, Xue Sun, Xifeng Wu, Zhenya Song

**Affiliations:** a Department of General Practice and Health Management Center, the Second Affiliated Hospital, School of Medicine, Zhejiang University, Hangzhou, China; b Department of Big Data in Health Science, School of Public Health, Zhejiang University, Hangzhou, China; c Center for Biostatistics, Bioinformatics, and Big Data, Second Affiliated Hospital, School of Medicine, Zhejiang University, Hangzhou, China

**Keywords:** Carotid plaque, Liver fibrosis, NAFLD fibrosis score, Non-alcoholic fatty liver disease

## Abstract

**Purposes:**

There is increasing concern regarding cardiovascular risk in non-alcoholic fatty liver disease (NAFLD) patients with liver fibrosis. This study aims: (1) to assess the association between NAFLD and liver fibrosis status and the development of carotid plaque (CP), and (2) to identify CP risk factors among general population with different baseline NAFLD and liver fibrosis status.

**Methods:**

This retrospective cohort study included 14,288 adult participants who went for regular health check-ups between 2014 and 2019, in one hospital in Zhejiang, China. NAFLD was diagnosed by abdominal ultrasound and the NAFLD fibrosis score (NFS) was calculated to reflect the extent of liver fibrosis. Cox proportional hazards analyses were applied to assess the risk of CP development across groups with different baseline NAFLD and NFS status.

**Results:**

NAFLD participants with high NFS had higher risk of CP compared to non-NAFLD participants (adjusted hazard ratio 1.68, 95% confidence interval [CI] 1.43–1.96, *p* < .001). Progression from NAFLD free and NAFLD with low NFS to NAFLD with high NFS are associated with 1.56-fold (95% CI 1.21–2.01, *p* = .001) and 1.43-fold (95% CI 1.11-1.84, *p* = .006) increased risk of CP, respectively. Risk factors associated with CP vary based on baseline NAFLD and NFS status. Among NAFLD participants with high NFS, hypertension is the only significant risk factor after adjustment for other potential influencing factors.

**Conclusions:**

NAFLD and liver fibrosis status can be an independent predictor for CP development regardless of metabolic abnormalities. Hypertension is a major risk factor for CP development among NAFLD patients with high NFS.

**KEY MESSAGES**
- Non-alcoholic fatty liver disease (NAFLD) and liver fibrosis status can be an independent predictor for development of carotid plaque.
- Progression from NAFLD free and NAFLD with low NAFLD fibrosis score (NFS) to NAFLD with high NFS are associated with increased risk of carotid plaque.
- Risk factors associated with carotid plaque vary based on baseline NAFLD and NFS status, and hypertension plays the most important role among patients with NAFLD and high NFS.

## Introduction

Non-alcoholic fatty liver disease (NAFLD) is the most common cause of chronic liver disease in the world, and its prevalence is up to 25.24% in the general population and over 76% in type 2 diabetics (T2DM) [1,2]. Notably, it has been recognised that cardiovascular disease (CVD) represents the most common cause of death of NAFLD (about 43%) [3]. The spectrum of NAFLD ranges from hepatic steatosis to non-alcoholic steatohepatitis, advanced fibrosis, cirrhosis and hepatocellular carcinoma [4]. The development and progression of liver fibrosis has been found to be the most important predictor of disease outcomes in NAFLD [3]. Furthermore, liver fibrosis is the independent predictor of cardiovascular morbidity and mortality in the general population [5,6]. Based on these findings, we have reason to speculate that NAFLD with fibrosis could be a major risk factor for CVDs.

Carotid plaque (CP), a lipid-driven inflammatory vascular disease, is a surrogate for subclinical CVD and is a predictor of cardiovascular events [7,8]. Accumulating epidemiological and clinical studies demonstrated that the incidence of CP increased in NAFLD individuals, which may be attributable to metabolic abnormalities such as hypertension, DM, and dyslipidemia; however, whether it is related to liver fibrosis was not completely clear [9,10]. There was paucity of studies on the effect of NAFLD with liver fibrosis on the development of subclinical atherosclerosis. Most previous related studies were cross-sectional or were limited to specific populations, providing insufficient evidence to determine a causal relationship in the general population [5,9,11–13]. One study based on small size sample found that liver fibrosis was not associated with increased carotid intima-media thickness (CIMT) [14], but overall, the impact of liver fibrosis on the development of CP was not well studied.

We hypothesised that NAFLD and liver fibrosis status are associated with the development of CP in the general population. To test this hypothesis, we performed a large retrospective cohort study among individuals who went for regular health check-ups. Based on health check-up results data, we examined factors that potentially contribute to the development of CP among people with different NAFLD and liver fibrosis status.

## Materials and methods

### Study population

**Figure 1.**
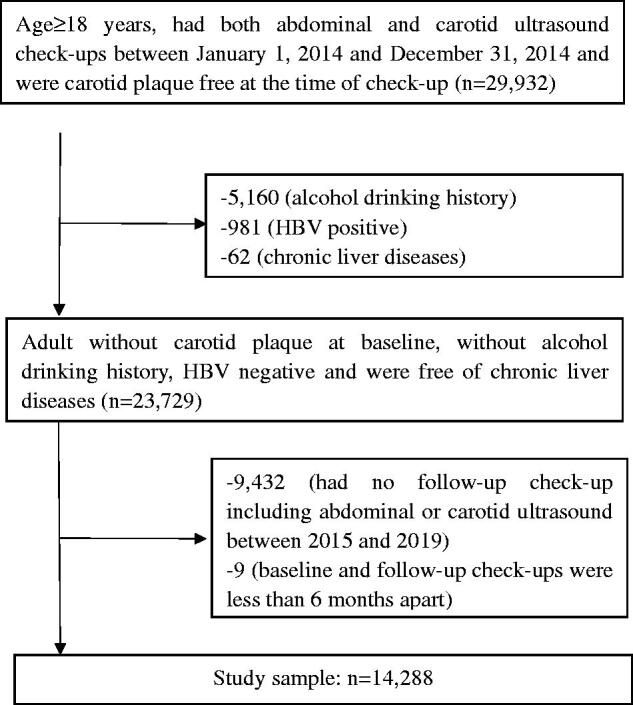
Flow diagram of study participants selection.

This retrospective cohort study included individuals who went for regular health check-ups between January 2014 and December 2019, in the Second Affiliated Hospital of Zhejiang University, School of Medicine, Hangzhou, Zhejiang, China. Inclusion criteria were as follows: age ≥ 18 years; had both abdominal and carotid ultrasound check-ups between 2014, January 1 and 2014, December 31 and were CP free; had at least one follow-up check-up including both abdominal and carotid ultrasound before 2019, December 31; the baseline and follow-up check-ups had to be at least 6 months apart. Subjects with any of the following conditions were excluded from this study: alcohol drinking history; hepatitis B virus positive; chronic liver diseases. The flow diagram for subject selection process is presented in Figure 1. This study was approved by the Institutional Review Board of the Second Affiliated Hospital of Zhejiang University, School of Medicine (2020–1110), with the requirement for informed consent waived. All study procedures were conducted according to the ethical guidelines of the 1975 Declaration of Helsinki.

### Measurements

Medical history of hypertension, DM, chronic liver diseases, personal history of drinking and smoking were systematically acquired by physicians. Blood pressure and anthropometric were measured as described in our previous study [15]. Venous blood samples were obtained in the morning after overnight fasting. Triglyceride (TG), total cholesterol (TC), low-density lipoprotein cholesterol (LDL-C), high-density lipoprotein cholesterol (HDL-C), alanine aminotransferase (ALT), aspartate aminotransferase (AST), gamma-glutamyl transferase (GGT), fasting plasma glucose (FPG), albumin, creatinine (Cr), C-reactive protein (CRP), and serum uric acid (sUA) were analysed using an Olympus AU4500 automatic chemistry analyser (Olympus, Tokyo, Japan). Glycosylated haemoglobin (HbA1c) was determined using an Arkray HA8160 automatic analyser (Arkray, Kyoto, Japan). Fasting insulin was measured using a Roche E170 auto-analyser (Roche, Mannheim, Germany). White blood cell (WBC) count and platelet count were measured with a Sysmex XN-9000 automatic haematology analyser (Sysmex, Kobe, Japan).

Carotid and abdominal ultrasound examinations were routinely performed in a health check-up at our health management centre by experienced ultrasonographers following a standardised protocol. High-resolution sonography machines (LOGIQ E9, GE Healthcare, Wauwatosa, WI, USA) were used for carotid ultrasound with a 10-MHz probe and for abdominal ultrasound with a 3.5-MHz probe. CIMT was measured the vertical distance from the upper edge of the intima to the upper edge of the adventitia on the far wall of the distal 10 mm of the common carotid artery. CP was defined as an IMT >1.5 mm in any portion of the carotid arteries [16]. Fatty liver was diagnosed based on known standard criteria, including the evidence of diffuse hyperechogenicity of the liver when compare to the kidney, vascular blurring, and deep attenuation of ultrasound signal [17].

### Definitions

NAFLD was diagnosed by abdominal ultrasonography after excluding subjects with alcohol consumption history and other causes of chronic liver disease, such as viral hepatitis, parenteral nutrition, medications, Wilson’s disease or severe malnutrition [18]. Among subjects who were classified as NAFLD, to reflect the extent of liver fibrosis, the NAFLD fibrosis score (NFS) was calculated as −1.675 + 0.037 * age (years) + 0.094 * body mass index (BMI) (kg/m^2^) + 1.13 * impaired FPG/DM (yes = 1, no = 0) + 0.99 * AST/ALT ratio − 0.013 * platelet count (× 10^9^/l) − 0.66 * albumin (g/dl) [19]. The NFS was further categorised into two levels: low NFS (<−1.455) and high NFS (≥ −1.455) [19].

According to the revised Adult Treatment Panel III and the Chinese Diabetes Society criteria [20,21], we used BMI as a surrogate for waist circumference to define metabolic syndrome (MetS) because waist circumference was not available in our data. The following criteria were applied to define the conditions of hypertension, DM, dyslipidemia, obesity and MetS:

- Hypertension: systolic blood pressure (SBP) ≥ 140 mm Hg, or diastolic blood pressure (DBP) ≥ 90 mm Hg, or previous diagnosis
- DM: FPG ≥ 7mmol/l or previous diagnosis
- Dyslipidemia: TG ≥ 1.7 mmol/L, or HDL-C < 1.04 mmol/L for male, or HDL-C < 1.29 mmol/L for female, or previous diagnosis
- Obesity: BMI ≥ 25 kg/m^2^ in Asians [22]
- MetS: having three or more of the following traits
  - Elevated TG: ≥ 1.7 mmol/L
  - Reduced HDL-C: < 1.04 mmol/L for male or < 1.29 mmol/L for female
  - Elevated FPG: ≥ 5.6 mmol/l
  - Elevated blood pressure: SBP ≥ 130 mm Hg or DBP ≥ 85 mm Hg
  - BMI ≥ 25 kg/m^2^

### Statistical analysis

The subjects’ characteristics by CP status at the end of follow-up was compared using independent sample *t*-test for continuous variables and chi-square test for categorical variables where appropriate. The Kaplan-Meier survival curves were constructed to visually compare the risk of developing CP across the groups of NAFLD free, NAFLD with low NFS and NAFLD with high NFS at baseline. The unadjusted Cox proportional hazards regression analysis was applied to provide an unadjusted estimation of CP risk differences over the aforementioned three groups. The analysis was further adjusted for metabolic abnormalities including hypertension, DM, obesity, and dyslipidemia. The interaction effect between baseline NAFLD and liver fibrosis status and each of the four metabolic abnormalities was also evaluated, respectively.

To assess whether the progression of NAFLD and liver fibrosis status has any effect on the development of CP, we categorised the study population into nine groups defined by each individual’s NAFLD and NFS status at baseline and at the end of follow-up, respectively. Within each baseline strata, the risk of CP over different degree of NAFLD and liver fibrosis progression (i.e. developed, regressed and persistent) were compared.

To identify factors associated with development of CP, and to assess whether those associated factors vary by baseline NAFLD and NFS status, three stepwise Cox proportional hazard analyses were performed. The significance level for entering was set at .05, and for removal was set at .1. Potential influencing factors for model selections included gender, smoking status, hypertension, DM, dyslipidemia, obesity, MetS and selected laboratory test results such as HbA1c, Cr, sUA and GGT. Factors reflected in NFS calculation (e.g. age) or through metabolic abnormalities were not included as individual variables during the model selection process in order to avoid multicollinearity. Three multivariable Cox proportional hazard models were constructed to assess the associations between identified prognostic factors and development of CP among people with non-NAFLD, with NAFLD and low NFS and with NAFLD and high NFS at baseline, respectively.

The proportional hazard assumption was tested for all Cox proportional hazard regression analyses and no violation was observed. *p*-Values less than .05 were considered statistically significant. All statistical analyses were performed using Stata 13.0 (StataCorp, College Station, TX).

## Results

The final analysis sample included 14,288 participants, among whom 2104 (14.72%) developed CP during follow-up. The median follow-up period was 4.00 years (range: 0.55–5.97 years). Participants who developed CP were relatively older, more likely to be male and smokers, had higher SBP, DBP, TC, TG, LDL-C, FPG, HbA1c, insulin, ALT, AST, GGT, platelet count, Cr, CRP and sUA, and lower HDL-C, albumin, and platelet count when compared to their CP free counterparts (Table 1). The prevalence of hypertension, DM, obesity, dyslipidemia and MetS were also higher in the CP group compared to the CP free group.

**Table 1.** Characteristics of study participants at baseline^†,‡^.

	All (*n* = 14,288)	Developed carotid plaque at the end of follow-up		*p* Value
		No (*n* = 12,184)	Yes (*n* = 2104)	
Age (years)	44.88 ± 11.46	43.14 ± 10.55	55.00 ± 11.27	<.001
Gender				
Male	7483 (52.37%)	6187 (50.78%)	1296 (61.60%)	<.001
Female	6805 (47.63%)	5997 (49.22%)	808 (38.40%)	
Smoking				
Yes	2239 (15.67%)	1766 (14.49%)	473 (22.48%)	<.001
No	12,049 (84.33%)	10,418 (85.51%)	1631 (77.52%)	
Obesity (BMI ≥ 25)				
Yes	3829 (27.38%)	3093 (25.87%)	736 (36.27%)	<.001
No	10,155 (72.62%)	8862 (74.13%)	1293 (63.73%)	
Hypertension				
Yes	3038 (21.26%)	2213 (18.16%)	825 (39.21%)	<.001
No	11,250 (78.74%)	9971 (81.84%)	1279 (60.79%)	
Diabetes				
Yes	554 (3.88%)	363 (2.98%)	191 (9.08%)	<.001
No	13,743 (96.12%)	11,821 (97.02%)	1913 (90.92%)	
Dyslipidemia				
Yes	6247 (43.72%)	5122 (42.04%)	1125 (53.47%)	<.001
No	8041 (56.28%)	7062 (57.96%)	979 (46.53%)	
MetS				
Yes	2904 (20.57%)	2200 (18.24%)	704 (34.27%)	<.001
No	11,212 (79.43%)	9862 (81.76%)	1350 (65.73%)	
FBG (mmol/L)	5.16 ± 0.82	5.11 ± 0.75	5.45 ± 1.09	<.001
HbA1c (%)	5.46 ± 0.53	5.42 ± 0.48	5.70 ± 0.70	<.001
SBP (mmHg)	121.21 ± 16.71	119.96 ± 16.26	128.56 ± 17.39	<.001
DBP (mmHg)	73.66 ± 11.28	73.02 ± 11.15	77.41 ± 11.30	<.001
LDL-C (mmol/L)	2.86 ± 0.74	2.82 ± 0.72	3.09 ± 0.77	<.001
HDL-C (mmol/L)	1.32 ± 0.33	1.33 ± 0.33	1.27 ± 0.32	<.001
Triglyceride (mmol/L)	1.53 ± 1.19	1.50 ± 1.17	1.74 ± 1.28	<.001
TC (mmol/L)	4.99 ± 0.92	4.94 ± 0.90	5.26 ± 0.97	<.001
ALT(U/L)	24.02 ± 18.60	23.82 ± 18.81	25.16 ± 17.28	.0012
AST(U/L)	23.53 ± 9.44	23.35 ± 9.57	24.60 ± 8.54	<.001
GGT (U/L)	26.81 ± 26.31	26.19 ± 25.75	30.43 ± 29.09	<.001
Albumin (μmol/L)	45.69 ± 2.64	45.74 ± 2.65	45.38 ± 2.56	<.001
Platelet count (10^9/L)	207.34 ± 51.00	208.07 ± 50.70	203.15 ± 52.51	<.001
Creatinine (μmol/L)	65.25 ± 15.10	64.73 ± 14.59	68.30 ± 17.50	<.001
CRP (mg/L)	1.12 ± 2.54	1.08 ± 2.48	1.47 ± 2.94	<.001
Uric acid (μmol/L)	332.21 ± 86.01	328.62 ± 85.74	352.99 ± 84.66	<.001
Insulin (pmol/L)	69.40 ± 41.39	69.00 ± 40.91	72.36 ± 44.76	.0038

^†^All values are presented as *n* (%) and mean ± standard deviation. Independent sample *t*-test for continuous variables and chi-square test for categorical variables.

^‡^ALT: alanine aminotransferase; AST: aspartate aminotransferase; BMI: body mass index; DBP: diastolic blood pressure; FPG: fasting plasma glucose; GGT: glutamyl transferase; HbA1c: glycosylated haemoglobin; HDL-C: high-density lipoprotein cholesterol; LDL-C: low-density lipoprotein cholesterol; MetS: metabolic syndrome; SBP: systolic blood pressure; TC: total cholesterol.

### Association between baseline NAFLD and NFS status and development of CP

**Figure 2.**
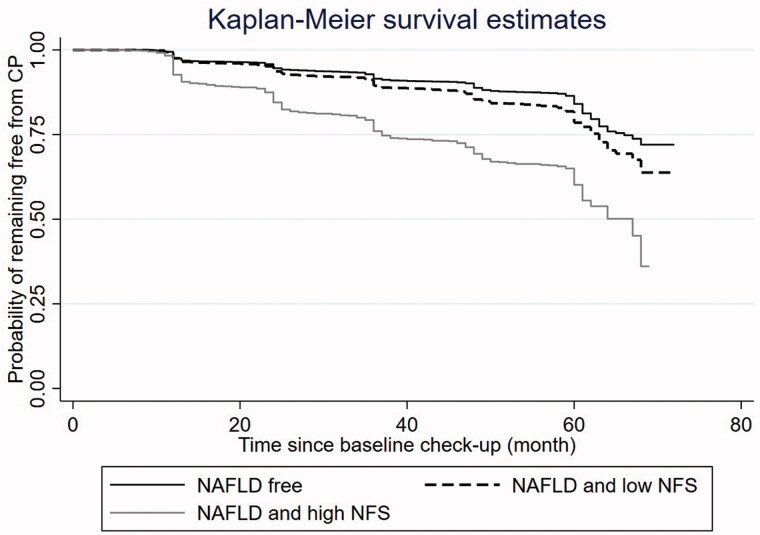
Kaplan-Meier curve of the risk of carotid plaque development by NAFLD and NFS status at baseline. NAFLD: Non-alcoholic fatty liver disease; NFS: NAFLD fibrosis score.

Among the 14,288 participants, 9214 (64.49%) were NAFLD free at baseline, 3826 (26.78%) had NAFLD but with low NFS, 922 (6.45%) had NAFLD with high NFS, and 326 (2.28%) had NAFLD with unknown NFS status due to lack of necessary data to calculate the score. The unadjusted analysis (Table 2) suggests that compared to the NAFLD free group, the risk of developing CP was 1.42-fold (95% confidence interval [CI] 1.29–1.57, *p* < .001) and 2.99-fold (95% CI 2.63–3.39, *p* < .001) higher in NAFLD/low NFS group and NAFLD/high NFS group, respectively. The Kaplan-Meier curves visually presented and compared the probability of remaining free from CP during follow-up across the three groups (Figure 2). After adjusting for gender, hypertension, DM, obesity, and dyslipidemia, the risk difference between the NAFLD/low NFS group and the NAFLD free group became insignificant; however, the NAFLD/high NFS group still had 1.68-fold (95% CI: 1.43–1.96, *p* < .001) increased CP risk compared to the NAFLD free group.

**Table 2.** Risk of carotid plaque development by NAFLD and NFS status at baseline**^†,‡^**.

Baseline status	Unadjusted HR (95%CI)	*p* Value	Adjusted HR (95% CI)	*p* Value
NAFLD free	Ref.	–	Ref.	–
NAFLD and low NFS	1.42 (1.29 − 1.57)	<.001	1.04 (0.92 − 1.17)	.544
NAFLD and high NFS	2.99 (2.63 − 3.39)	<.001	1.68 (1.43 − 1.96)	<.001

**^†^**Adjusted for gender, hypertension, diabetes, obesity and dyslipidemia. Age was not adjusted since it was included in the calculation of NFS scores.

**^‡^**CI: confidence interval; HR: hazard ratio; NAFLD: Non-alcoholic fatty liver disease; NFS: NAFLD fibrosis score.

Further analyses including interaction terms between the baseline NAFLD/NFS status and each of the four metabolic abnormalities (*p* values for interaction between baseline NAFLD/NFS status and metabolic abnormalities were less than .001 for hypertension, obesity, and DM, respectively, and was .093 for dyslipidemia) indicate that development of CP was significantly associated with the interaction effect, suggesting necessity for stratified analysis.

### Association between progression of NAFLD and NFS status and development of CP

Among those who were NAFLD free at baseline, the incidence of CP among subgroups that remained NAFLD free, developed NAFLD with low NFS and developed NAFLD with high NFS during follow-up were 11.08%, 13.36% and 22.03%, respectively (Table 3). For NAFLD free individuals at baseline, developing NAFLD with high NFS during follow-up was associated with 1.56-fold (95% CI 1.21–2.01, *p* = .001) increased risk of CP; However, if the progression was only limited to NAFLD with low NFS, the risk increase was not statically significant (hazard ratio [HR] 1.04, 95% CI 0.88 − 1.22, *p* = .678).

**Table 3.** Risk of carotid plaque development by NAFLD and NFS status at baseline and follow-up^†^.

Baseline	Follow-up	All *n* (%)	Carotid plaque *n* (%)	HR (95% CI)	*p* Value
NAFLD free	NAFLD free	7,601 (82.97%)	842 (11.08%)	Ref.	–
	NAFLD and low NFS	1,265 (13.81%)	169 (13.36%)	1.04 (0.88 − 1.22)	.678
	NAFLD and high NFS	295 (3.22%)	65 (22.03%)	1.56 (1.21 − 2.01)	.001
NAFLD and low NFS	NAFLD and low NFS	2,884 (76.91%)	449 (15.57%)	Ref.	–
	NAFLD free	584 (15.57%)	102 (17.47%)	1.16 (0.94 − 1.44)	.165
	NAFLD and high NFS	282 (7.52%)	69 (24.47%)	1.43 (1.11 − 1.84)	.006
NAFLD and high NFS	NAFLD and high NFS	576 (63.58%)	191 (33.16%)	Ref.	–
	NAFLD free	165 (18.21%)	56 (33.94%)	0.96 (0.71 − 1.29)	.768
	NAFLD and low NFS	165 (18.21%)	43 (26.06%)	0.82 (0.59 − 1.14)	.230

**^†^**CI: confidence interval; HR: hazard ratio; NAFLD: non-alcoholic fatty liver disease; NFS: NAFLD fibrosis score; Ref.: Reference.

Among subjects with NAFLD and low NFSs, those with elevated levels of NFS during follow-up had 1.43-fold (95% CI 1.1–1.84, *p* = .006) increased risk of CP compared to those with NAFLD but had persistent low NFS. Regression of NAFLD/low NFS status to NAFLD free status did not suggest significant reduced risk of CP. For people who were already NAFLD with high NFS at baseline, regression of the liver status to low NFS or NAFLD free status did not significantly reduce the risk of CP.

### Factors associated with CP development by baseline NAFLD and liver fibrosis status

According to the stepwise model selection analysis results, factors associated with CP development varied across different baseline NAFLD and liver fibrosis status. The multivariable Cox proportional hazard regression analysis results were presented in Table 4.

**Table 4.** Factors associated with development of carotid plaque by baseline NAFLD and NFS status^†,‡^.

	Model I (NAFLD free)		Model II (NAFLD and low NFS)		Model III (NAFLD and high NFS)	
	HR (95% CI)	*p* Value	HR (95% CI)	*p* Value	HR (95% CI)	*p* Value
Hypertension						
No	Ref.		Ref.		Ref.	
Yes	2.50 (2.17–2.88)	<.001	1.52 (1.28–1.82)	<.001	1.47 (1.16–1.85)	.001
Dyslipidemia						
No	Ref.		–	–	–	–
Yes	1.23 (1.08–1.40)	.002	–	–	–	–
Smoking						
No	Ref.		Ref.		–	–
Yes	1.34 (1.12–1.61)	.001	1.34 (1.11–1.62)	.003	–	–
HbA1c (%)						
Q_1_ (≤5.2)	Ref.		Ref.		–	–
Q_2_ (5.3–5.4)	1.54 (1.28–1.86)	<.001	1.18 (0.89–1.58)	.243	–	–
Q_3_ (5.5–5.6)	2.07 (1.71–2.50)	<.001	1.65 (1.26–2.16)	<.001	–	–
Q_4_ (≥5.7)	3.30 (2.76–3.96)	<.001	1.95 (1.51–2.50)	<.001	–	–
Creatinine (μmol/L)						
Q_1_ (≤53)	Ref.		Ref.		Ref.	
Q_2_ (54–64)	1.17 (0.97–1.40)	.096	1.08 (0.80–1.46)	.617	1.38 (0.94–2.04)	.103
Q_3_ (65–75)	1.15 (0.93–1.42)	.189	0.99 (0.70–1.40)	.939	1.18 (0.80–1.74)	.400
Q_4_ (≥76)	1.31 (1.05–1.63)	.016	1.16 (0.81–1.66)	.413	1.36 (0.94–1.96)	.099
Uric acid (μmol/L)						
Q_1_ (≤267)	Ref.		–	–	–	–
Q_2_ (268–325)	1.12 (0.93–1.33)	.228	–	–	–	–
Q_3_ (326–388)	1.24 (1.02–1.52)	.035	–	–	–	–
Q_4_ (≥389)	1.37 (1.09–1.72)	.007	–	–	–	–
Male						
No	–	–	Ref.		–	–
Yes	–	–	0.91 (0.68–1.21)	.508	–	–
Obesity and/or MetS						
Neither	–	–	Ref.		–	–
MetS without obesity		–	1.41 (1.08–1.84)	.011	–	–
Obesity without MetS		–	0.74 (0.57–0.97)	.031	–	–
Both	–	–	1.04 (0.85–1.28)	.693	–	–

**^†^**Age was not adjusted since it was included in the calculation of NFS scores.

**^‡^**CI: confidence interval; HbA1c: glycosylated haemoglobin; HR: hazard ratio; MetS: metabolic syndrome; NAFLD: Non-alcoholic fatty liver disease; NFS: NAFLD fibrosis score; Q: quartile; Ref.: Reference.

For people who are NAFLD free at baseline, hypertension (HR 2.50, 95% CI 2.17–2.88, *p* < .001), dyslipidemia (HR 1.23, 95% CI 1.08–1.40, *p* = .002), smoking (HR 1.34, 95% CI 1.12–1.61, *p* = .001), elevated levels of HbA1c (2nd quartile (Q2) [5.3–5.4%] vs Q1 [≤5.2%], HR 1.54, 95% CI 1.28–1.86, *p* < .001; Q3 [5.5–5.6%] vs Q1 [≤5.2%], HR 2.07, 95% CI 1.71–2.50, *p* < .001; Q4 [≥5.7%] vs Q1[≤5.2%], HR 3.30, 95% CI 2.76–3.96, *p* < .001) , Cr (Q4 [≥76 μmol/L] vs Q1 [≤53 μmol/L], HR 1.31, 95% CI 1.05–.63, *p* = .016) and sUA (Q3 [326 − 388 μmol/L] vs Q1 [≤267 μmol/L], HR 1.24, 95% CI 1.02–1.52, *p* = .031; Q4 [≥389 μmol/L] vs Q1 [≤267 μmol/L], HR 1.37, 95% CI 1.09–1.72, *p* = .007) were associated with increased risk of CP.

For the group with NAFLD and low NFS at baseline, participants with hypertension, who are smokers and with elevated HbA1c levels have increased risk of CP. Cr and gender were also included in the model based on stepwise model selection results; however, the association between the two factors and CP risk were not significant after adjusting for other selected variables. Obesity and MetS were also found to be predictors of CP. Further analysis indicated that the interaction effect between the two factors was significantly associated with the development of CP. Among people with NAFLD and low NFS, compared to people with neither obesity nor MetS, those with MetS but no obesity had 1.41-fold (95% CI 1.08–1.84, *p* = .011) increased risk of CP; however, those with obesity only but no MetS had 0.26-fold decreased risk of CP (95% CI 0.57–0.97, *p* = .031).

Among people who already had NAFLD with high NFS, the risk of CP development was significantly associated with hypertension (HR 1.47, 95% CI 1.16–1.85, *p* = .001). Cr level may be a potential influencing factor; however, the risk of CP over different Cr quartile levels did not differ significantly after adjusting for hypertension status.

## Discussion

In this large retrospective cohort study focussing on assessment of the association between NAFLD and liver fibrosis status and the risk of CP, we found that participants with NAFLD and high NFS were at higher risk of developing CP compared to those without NAFLD, even after adjusting for gender and metabolic abnormalities, including, obesity, hypertension, DM, and dyslipidemia. Our results also show that progression of non-NAFLD to NAFLD with high NFS, or progression of NAFLD with low NFS to NAFLD with high NFS was associated with higher risk of developing CP; however, regression of NAFLD or change from high NFS to low NFS during follow-up did not significantly reduce the risk of CP development. Furthermore, we found that hypertension plays a more important role compared to other factors of interests and is the major risk factor for CP development among participants with NAFLD and high NFS. To our knowledge, this is the first study about the effect of NAFLD and liver fibrosis on CP development based on a large healthy cohort among Chinese population.

Epidemiological studies and meta-analysis have found a close association between NAFLD and subclinical atherosclerosis and CVD [9,12,23,24]. NAFLD is considered as hepatic manifestation of the MetS, and increasing evidence suggested that NAFLD may be both a cause and a consequence of MetS and its components, with insulin resistance (IR) as a common pathophysiological mechanism [25,26]. NAFLD and MetS can be considered to have similar effects on contribution to accelerated atherogenesis, possibly *via* chronic low-grade inflammation, atherogenic dyslipidemia, increased oxidative stress, unbalanced coagulation-fibrinolysis, and adipokines imbalance [26].

In our study, we found that development of CP within a 5-year follow-up period occurred more frequently in subjects with NAFLD and high NFS, further adjustment for gender, hypertension, DM, obesity, and dyslipidemia did not alter the significance of this association. It suggests that liver fibrosis may serve as a predictive marker for increased susceptibility to CP development regardless of metabolic abnormalities. These results were concordant with the findings of previous studies. A cross-sectional study including 400 individuals in Italy found that individuals at high probability of fibrosis had a 3.9-fold increased risk of vascular atherosclerosis as compared with individuals at low probability of fibrosis [5]. A longitudinal cohort study on 1120 T2DM showed that hepatic steatosis with fibrosis is independently associated with the progression of carotid atherosclerosis [12].A retrospective cohort study on 3,185 adult men without carotid atherosclerosis at baseline showed that NAFLD patients with a high NFS and fibrosis-4 scores at baseline had higher risk of subclinical carotid atherosclerosis development [9], however, this finding is only generalisable to male population. A cross-sectional study with 251 subjects in Malaysian showed that NAFLD and advanced liver fibrosis assessed using FibroScan appeared to be not associated with increased CIMT [14]; however, given the small sample size, the result may be affected by insufficient statistical power.

We found no incremental risk of CP in participants with NAFLD but low NFS at baseline compared with those who are NAFLD free. Moreover, progression from non-NAFLD to NAFLD with low NFS during follow-up did not significantly increase the risk of CP. These findings were consistent with previous related studies. According to a long-term, clinical study with 20.4-year follow-up, patients with simple hepatic steatosis were more likely to follow a relatively benign course and have a good prognosis, with mortality similar to the general population [27].A narrative review and clinical perspective of prospective data have suggested that NAFLD uncomplicated by steatohepatitis or fibrosis is insufficient to be considered as high risk for CVD [28]. A longitudinal cohort also found that hepatic steatosis without fibrosis was not statistically significantly associated with CP progression in T2DM [12].

In our study, among the potential risk factors we examined, hypertension has consistently to be found as a risk factor for all participants regardless of their baseline NAFLD and NFS status. Furthermore, among participants with NAFLD and high NFS, hypertension is the only risk factor we found to be significantly associated with CP after adjusting for metabolic factors, suggesting the potential of controlling CP risk through intervening on blood pressure, especially among individuals with NAFLD and high NFS. Blood pressure has a continuous and consistent relationship with the risk of incident atherosclerosis, and hypertension is one of the strongest risk factors for CP [29]. There is growing evidence suggesting that NAFLD may be both a consequence and a cause of hypertension [26,30,31]. A cross-sectional analysis of 11,489 adults from the 2005 to 2016 National Health and Nutrition Examination Survey revealed that hypertension patient showed a higher prevalence of advanced fibrosis, and a continuous relationship exists between advance fibrosis and blood pressures [32]. A prospective study with 149 biopsy-proven NAFLD obese patients found that hypertension was independent predictive factors of worsening fibrosis [33]. A recent meta-analysis of 11 cohort studies including 411 patients with biopsy-proven NAFLD showed that hypertension almost doubled the risk of the development of progressive fibrosis [34]. A possible mechanism is through increased IR and decreased interleukin −10-mediated or haem oxygenase −1-induced anti-inflammatory mechanisms [35]. Prospective studies are needed to determine whether treatment and management of hypertension for NAFLD with fibrosis patients can reduce the risk for the development of CP.

In addition, we found that smoking and elevated HbA1c level were associated with increased risk of CP development among participants without NAFLD or with NAFLD but low NFS. Smoking is an important modifiable risk factor for the development of CVD and progression of atherosclerosis [36,37]. Smoking leads to atherosclerosis progression by numerous mechanisms, including increased lipid peroxidation [38], endothelial dysfunction and microvascular damage [39], proliferation of vascular smooth muscle cells [40], and formation of macrophage foam cells [41]. Previous studies have found that elevated HbA1c was a significant predictor of CVD mortality and was an independent risk factor for subclinical atherosclerosis, the mechanism may be related to IR and glucose excursion [42–44]. Besides, among NAFLD with low NFS participants, we found that compared to subjects with neither obesity nor MetS, obese participants without MetS had 0.26-fold decreased risk of CP; however, the risk was 1.41-fold higher in nonobese participants with MetS. Several previous studies have proven the existence of a metabolically benign obese phenotype that displays a lower risk of atherosclerosis than the metabolically obese normal-weight phenotype [45–47]. Non-obese NAFLD may represent a subset phenotype of NAFLD in normal-weight but metabolically obese subjects due to the well-known association between IR and NAFLD [48]. Recently, a cohort study also found that lean NAFLD patients have a higher overall mortality than overweight or obese NAFLD patients over an average of 11-year follow-up [49]. Based on our findings, among people with NAFLD and low NFS, those non-obese individuals with MetS may need specific attention regarding potential excessive risk of CP.

In our study, we found that progression from NAFLD free to NAFLD with low NFS does not significantly increase the risk of development of CP, we speculate that the elevation of risk of CP is most likely to happen during the period when the status of NAFLD with low NFS gradually progressed to the status of NAFLD with high NFS. In other words, when subjects are in NAFLD status, their risk of CP started to elevate when their NFS started to increase from a certain threshold. Also, there could possibly be another upper threshold, and once exceeded, the risk of CP will remain unchanged. The dose-response effect as well as the potential thresholds could be further explored by further studies.

Our study has some limitations. First, a non-invasive diagnostic scoring system, rather than liver biopsy, was used to assess the extent of liver fibrosis, which is not the gold standard for the diagnosis of liver fibrosis. However, NFS has high prediction accuracy and has been guideline-recommended to be used for non-invasive diagnosis of liver fibrosis [19,50]. Second, instead of CVD events which could represent a direct cardiovascular outcome of NAFLD, CP by ultrasonography as the sign of carotid deterioration was analysed in this study. Due to data limitation, investigation of cardiovascular events, as well as effects of other potential influencing factors, such as diet, exercise, and medication, was not allowed in this study. Third, ultrasound has lower sensitivity and specificity on diagnosis of CP morphology than computerised tomography angiography and magnetic resonance angiography [51]. However, it is globally accepted as the standard imaging examination for first-line diagnosis of carotid atherosclerosis, at least for screening, because of rapidly applicable, readily available, relatively low cost, and non-invasive technique [51]. Fourth, although it is noted that both vascular disease and NAFLD have sexual dimorphism [52,53], we found that gender is not a significant influencing factor for development of CP in the subgroups of NAFLD free and NAFLD with high NFS group. Additionally, since our analyses were stratified by the NAFLD status, we speculate that the gender effect associated with the NAFLD status might have been diluted due to the stratification. Furthermore, due to the incomplete and lack of reproductive information in the health check-up data, discussion about potential effect of reproductive history on development of CP was not available in this study. Finally, this study was based on a single centre cohort of Chinese population recruited from a Health Management Centre. Further multicenter prospective studies are needed to validate the results, as well as to assure the generalisability of the study findings.

There are also several strengths in our research that should be highlighted. On the one hand, our study was a retrospective cohort study including comparatively large sample size (14,288 participants) with sufficient follow-up period (5 years). On the other hand, our study was performed in general population which are representative for healthy population without specific health concern.

In summary, our findings suggest that NAFLD and liver fibrosis status can be an independent predictor for CP even after adjusting for gender and metabolic abnormalities. Progression of NAFLD and fibrosis status during follow-up may increase the risk of CP. Risk factors for CP varied by baseline NAFLD and fibrosis status, and hypertension plays the most important role for development of CP among subjects with NAFLD and high NFS. The CP risk factors we identified among people with different NAFLD and liver fibrosis status were completely based on regular health check-up results, indicating that effective evaluation of risk of CP development during regular health check-ups are clinically practicable. Moreover, tailored intervention strategies for reducing CP risk could be developed given patients’ baseline NAFLD and liver fibrosis status and to target on specific risk factors varied by the baseline status.

## Data Availability

The data that support the findings of this study are available from the corresponding author, [Zhenya Song], upon reasonable request.
